# A social cognition perspective on misophonia

**DOI:** 10.1098/rstb.2023.0257

**Published:** 2024-08-26

**Authors:** Joel I. Berger, Phillip E. Gander, Sukhbinder Kumar

**Affiliations:** ^1^ Department of Neurosurgery, University of Iowa, Iowa City, 52242 USA; ^2^ Department of Radiology, University of Iowa, Iowa City, 52242 USA

**Keywords:** action perception, auditory, psychology, theoretical

## Abstract

Misophonia is commonly classified by intense emotional reactions to common everyday sounds. The condition has an impact both on the mental health of its sufferers and societally. As yet, formal models on the basis of misophonia are in their infancy. Based on developing behavioural and neuroscientific research we are gaining a growing understanding of the phenomenology and empirical findings in misophonia, such as the importance of context, types of coping strategies used and the activation of particular brain regions. In this article, we argue for a model of misophonia that includes not only the sound but also the context within which sound is perceived and the emotional reaction triggered. We review the current behavioural and neuroimaging literature, which lends support to this idea. Based on the current evidence, we propose that misophonia should be understood within the broader context of social perception and cognition, and not restricted within the narrow domain of being a disorder of auditory processing. We discuss the evidence in support of this hypothesis, as well as the implications for potential treatment approaches.

This article is part of the theme issue ‘Sensing and feeling: an integrative approach to sensory processing and emotional experience’.

## 1. Introduction

Misophonia is a disorder characterized by an intense emotional reaction and often physical discomfort to everyday sounds, such as other people eating or drinking, or nasal and throat sounds [1–3]. Although the condition was named and referred to in the early 2000s [4,5], scientific research into the condition has only begun in recent years. Based on the limited evidence available, the prevalence of clinically relevant misophonia is suggested to be anywhere from 5 to 18% [6–8], indicating its wide prevalence and a need for further research. Misophonia can lead to severe social isolation and withdrawal from familial activities [9], and it is highly distressing to those who experience it on a frequent basis, even leading to suicidal thoughts in up to approximately 20% of sufferers [10].

At present, neurophysiological or psychological models of misophonia are in their infancy, partly owing to a lack of research in the area until recent years. The earliest formal model of misophonia was put forward by Jastreboff and Jastreboff [11] and updated recently [12]. This model proposes that the negative emotional reaction in misophonia is caused by the trigger sounds, which provoke processing in auditory brain regions that then results in aberrant activity in brain areas involved in emotion processing. Importantly, this model makes a distinction between hyperacusis and misophonia: while the negative reaction in hyperacusis (increased sense of loudness discomfort) depends on acoustic features such as the spectral energy of the sound, misophonia is affected by the acoustic pattern and meaning, not on the intensity of the sound. It was, therefore, suggested that the negative reaction in misophonia was not primarily owing to abnormal processing in auditory pathways, as may be the case for hyperacusis, but instead related to aberrant processing in the ‘limbic’ system, which processes emotion-related information of the sound. While this model in its latest iteration does highlight that the emotional reaction in misophonia depends on the context—for example, a sound from a particular person may be a trigger in one place but not in another situation or place—the sound itself remains the most important component in driving the emotional reaction. Framing misophonia in a sociological context, a recent article by Noreña [13] does not focus on the sound *per se* and argues for a specific role of social conditioning and conformity in driving the existence of misophonia [13], relating the condition to expected social norms. However, thus far, no models of misophonia have considered how the neural processes related to social cognition may play a critical role. Here, we consider current evidence in support of a model of misophonia that we propose is centred around neural processes related to social cognition. Some of these ideas were initially presented in our previous work [14], but here we expand upon them in a formalized model and update with evidence published since that work.

## 2. Factors to consider for a model of misophonia

### (a) The source of the sound is an important factor in triggering reactions

There are a number of studies showing that the source of sound is important for setting the context within which sound is perceived. For example, Reuter & Oehler [15] presented two sounds: fingernails scratching on a chalkboard and the sound of squeaking chalk on a slate, which are typically perceived as aversive by most people [16], and asked a group of participants to rate the aversiveness of the sounds. Half of the participants were told the true source of the sounds and the other half were made to believe that the sounds were part of contemporary music. It was found that the same sounds were rated as less aversive and evoked a lower skin conductance response by the ‘musical-source’ group of participants than the ‘chalkboard-source’ group, indicating that belief regarding the source of sounds shapes the perception and physiological response to aversive sounds. In a similar experiment, Samermit *et al*. [17] asked participants to rate the discomfort and unpleasantness of typical aversive sounds, such as a knife grating on glass, when these were presented alone or in association with a silent video. The silent video could either be of the original source of the sound or a video source with positive attributes (e.g. silent video of bird chirping presented with the sound of a knife grating on glass). Importantly, the video source with positive attributes was temporally synchronized with the sound, so that the sound was likely to be attributed to the visual source. The results showed that sounds that were synchronized to videos with positive attributes were rated as less unpleasant, caused less discomfort and reduced the intensity of bodily sensations, again indicating that the perceived source of a sound can modulate the reaction to that sound. A similar finding was demonstrated by Cox [18] using static images, highlighting that biasing towards a particular expected context, without the temporal dynamics that videos include, can influence the reaction to a perceived sound.

In relation to misophonia, recent work shows that the source of a sound plays a crucial role in determining the reaction triggered by the sound [19–21]. These studies highlight that when the source of trigger sounds is correctly identified, participants with misophonia rate these sounds as more aversive than incorrectly identified sounds. Furthermore, non-eating sounds incorrectly identified as human eating sounds are rated as more aversive than correctly identified non-eating sounds [19]. The importance of social context has also been highlighted in the recent consensus definition of misophonia [22]. All of these studies highlight an important implication for understanding misophonia: the *perceived* source of a sound is important for whether and to what degree a particular sound is experienced as a trigger sound, even in cases of incorrectly identified sound sources, emphasizing that the sounds *per se* are not the driving factor in misophonia.

### (b) Trigger sounds recruit involvement of insular cortex

Previous models of misophonia have suggested that activation of ‘limbic’ areas of the brain by trigger sounds could be an important factor in the trigger reaction in misophonia [12,23]. Although the term ‘limbic’, in general, has been criticized [24,25] for being vague and often contradictory in its description of the emotional system, it typically includes brain structures like the amygdala, hippocampus, thalamus, hypothalamus, basal ganglia and cingulate gyrus. From the few neuroimaging studies of misophonia that exist, there is only limited evidence for the involvement of commonly defined ‘limbic’ structures, such as increased activation to trigger sounds in the anterior cingulate cortex in a single study [26] and structural abnormalities in the amygdala in participants with misophonia [27], although notably Schröder *et al*. [26] did not find any differences in amygdala activation between misophonia and control participants.

Contrastingly, a brain structure that has been more consistently found to be involved in misophonia studies is the anterior insula, a brain region often implicated in interoceptive awareness and social cognition. In one of the first fMRI studies of misophonia, Kumar *et al*. [28] showed stronger activation of the anterior insula specifically for trigger sounds in participants with misophonia. Furthermore, the anterior insula had stronger functional connectivity to areas of the default mode network (referring to brain areas active when not engaged in an explicit task), including ventromedial prefrontal cortex (vmPFC) and posterior cingulate cortex. The finding of involvement of the anterior insula has been supported in other fMRI studies [14,26,29]. Although the precise role of the anterior insula in misophonia remains unknown, there is now a large body of evidence that links the anterior insula to social processing in general and processing of social emotions in particular (for a review, see [30]). Social emotions refer to processes that are generated because of interactions with other people. For example, the anterior insula is activated not only by the experience of self-emotions but also by observing the emotional expressions of others [31,32]. In addition to the processing of social emotions, the anterior insula is involved in social pain experienced after social rejection [33], social compliance [34] and recognition of social cues [35]. It should be noted, however, that the specific role of the insula may include other relevant factors to misophoniau because the structure is also involved in processes beyond social cognition, such as high-level emotional processing including subjective feelings and awareness [36] and interoception [37,38]. Nonetheless, given the imaging evidence, models of misophonia should consider the potential role of the anterior insula.

### (c) Trigger sounds cause hyperactivation of motor cortex in misophonia

In addition to the insula, as highlighted in the previous section, another brain region with an as yet unclear role that has been shown to have stronger activation to trigger sounds in participants with misophonia is the motor cortex. Kumar *et al*. [14] showed stronger activation of ventral premotor cortex (vPMC) in misophonia participants, specifically in response to trigger sounds. One might argue that the activation of motor cortex in misophonia is owing to the motor component of the emotional response, for example, stronger facial muscle activation owing to feelings of anger/disgust, in response to trigger sounds. However, stronger connectivity of the motor cortex to both visual and auditory cortex in the resting state, i.e. when no explicit stimuli were presented, does not support this argument [14]. Using a small sample of participants, Hansen *et al*. [29] found increased connectivity between motor cortex and auditory cortex (as well as insula), although in their study this was not selective for orofacial motor cortex, a finding that could reflect the mild nature of misophonia experienced by their participants, as opposed to the more severe misophonia experienced by participants in Kumar *et al*. [14]. Thus, involvement of motor cortex in mediating responses to trigger sounds should be considered as a factor in misophonia.

### (d) Auditory cortex is not activated differently in misophonia compared to controls

When considering the pathogenesis of misophonia, it is tempting to interpret the condition as a disorder of sound perception. Indeed, even the origin of the word misophonia is derived from the Greek words for ‘hate’ and ‘sound’, although it is worth acknowledging that a literal interpretation of this definition as a basis for misophonia should be avoided [12]. Nonetheless, one would intuitively expect that there would be differential activation of the auditory cortex in misophonia compared to controls. Contrasting with this idea, the fMRI study by Kumar *et al*. [28] showed no differences in auditory cortex activation to trigger sounds between misophonia sufferers and controls. A recent study utilizing auditory brainstem responses to examine the integrity of the subcortical auditory pathway also found no differences between misophonia participants and controls [39]. It should be noted that the results of Kumar *et al.* [28] seemingly contrast with those of Schröder *et al*. [26], wherein activity in the posterior portion of the right superior temporal cortex was increased in participants with misophonia. However, in Schröder *et al*. [26] the increased activation did not occur in primary auditory cortex but in a higher-order area, specifically the lateral aspect of the posterior superior temporal gyrus/sulcus, which is known to be a multi-modal area activated by moving visual stimuli and involved in social perception [40]. Thus, the lack of differential activation indicates that trigger sounds are not processed differently in core auditory cortex in participants with misophonia when compared to controls.

### (e) A high prevalence of mimicry in misophonia

Potentially the strongest behavioural evidence that cannot be understood with the existing conceptual framework of misophonia comes from reports of a high incidence of mimicry in misophonia sufferers. Even in some of the earliest published case studies of misophonia, mimicry was mentioned as an important feature of the condition [9]. Early self-report evidence suggested an incidence of mimicry in approximately 55% of misophonia sufferers, although the sample size was small (*n* = 11; [1]). Recent evidence from a large-scale online study (*n* = 676) found a similar incidence of mimicry in those with misophonia (greater than 45%; [41]). Moreover, the study showed that (i) the tendency to mimic increased with increasing misophonia severity, (ii) compared to environmental sounds and other human-produced sounds, orofacial sounds (eating and chewing) were more likely to result in mimicking behaviour, and (iii) mimicry provided some relief from distress to individuals with misophonia. Why would participants with misophonia mimic in response to trigger sounds, and why does this act often provide relief when used as a coping strategy? These questions should be considered in a theoretical and conceptual model of misophonia.

## 3. Misophonia: from sound perception to action perception

Using an integrated framework where both trigger sounds and their social source are considered together, a different picture of misophonia emerges. Since the most common trigger sounds, for example someone eating/chewing, are associated with an action (e.g. orofacial movement) of another person, it could be the case that misophonic distress is owing to the perceived action of others and not owing to the sound *per se*, which is a by-product of that action. There is now overwhelming evidence that the psychological/neural processes involved in social signals are different compared with those for non-social signals (for reviews, see [42,43]). In social cognition and neuroscience, it is well known that the mere observation or hearing the sounds of others' actions leads to ‘mirroring’ or ‘mimicking’ of the same actions by the perceiver, without any intention or awareness to do so [44–46]. In this scenario, mimicry is commonly understood within the framework of a ‘perception–action’ link, which posits that perceiving the action of others automatically activates representations of that action in the perceiver that, in turn, causes movement execution that is congruent with the perceived actions. With respect to the underlying brain function, the perception–action link is suggested to be instantiated as communication between sensory areas and motor areas of the brain. Thus, with emphasis on the actions of the trigger-person, could it be that the perception–action link is stronger in misophonia, which is activated by the sight or sounds of an action? This would appear to be supported by the results of Kumar *et al.* [14], showing stronger resting state connectivity of both auditory and visual cortex to motor cortex for participants with misophonia compared with control participants. Moreover, the same study also showed stronger activation of the motor cortex in participants with misophonia specifically in response to trigger sounds. However, whether stronger resting state connectivity and activation reflect mirroring of an action or result from heightened auditory imagery, as for example occurs in professional musicians [47], needs further investigation.

The phenomenon of mimicry has been commonly studied within a social perception/cognition framework. Thus, given the high incidence of mimicry in misophonia highlighted above, it is useful to examine the role of imitation and mimicry within the wider social cognition literature. Non-conscious mimicry can often occur in a social setting, involving matching of others' behaviours, such as particular mannerisms or gestures of those with whom the mimicker is interacting. This type of mimicry is termed the ‘chameleon effect’, and has been shown to potentially increase liking between individuals and facilitate smoother interactions [48], although the specific effects of mimicry can depend on the social context in which it takes place [49], or even the specific orientation of the mimicked actions [50]. The chameleon effect has been shown to be stronger in people who show higher empathy [48]. The main types of empathy can be broadly defined as cognitive, affective and motor [51], which may have distinct neural correlates [51,52]. The specific role of empathy within misophonia is yet to be explored. Research examining this may elucidate whether mimicry potentially reflects other heightened cognitive processes in these individuals, or alternatively if it is a byproduct of the underlying neural mechanisms of misophonia, such as a coping strategy that inhibits a hyperactive insula. Interestingly, the anterior insula, which is particularly hyperactive in misophonia, has been long implicated as a neurobiological marker of empathy (e.g. [53–55]). Therefore, another possibility of hyper-activation in the insula is that it reflects a greater degree of a specific type of empathy in individuals with misophonia. As yet, a role for differing degrees of a particular subtype of empathy in misophonia has not been tested, though it is worth noting that the concept of motor empathy—the tendency to automatically mimic the motor expressions of others—may be the specific domain implicated, rather than necessarily the cognitive or affective constructs of empathy.

There is already neuroimaging evidence highlighting regions that may underlie the importance of mirroring and the role of context in misophonia. As mentioned above, early auditory areas such as primary auditory cortex do not differ between misophonia sufferers and controls [14,26,28]. The earliest level in the auditory hierarchy that appears to show any difference in misophonia sufferers is in the posterior superior temporal region [26]. Importantly, as well as being multi-modal, this area is also commonly implicated in the imitation of others, through a proposed perception–action link mechanism [56]. When combined with evidence of the high incidence of mimicry in misophonia, it is suggestive of the possibility that ‘hyper-mirroring’ may underlie the presence of the condition, as proposed by Kumar *et al*. [14].

**Figure 1.**
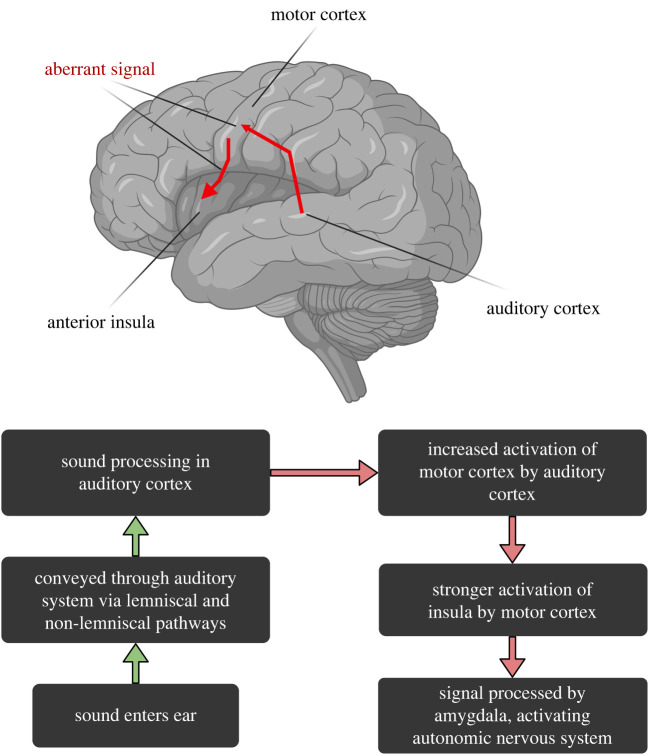
Simplified outline of the proposed model. Information about sounds travels from the ear through lemniscal and non-lemniscal auditory pathways, to be processed by higher level auditory cortex. Additionally, this signal is transmitted to motor regions to form a motor representation, which in the case of trigger sounds in misophonia is conveyed by an aberrant signal to the insula, causing hyperactivation of this region. The insula in turn communicates this to the amygdala, causing an emotional response and heightened autonomic processes (e.g. increased heart rate). Red arrows indicate parts of the pathway along which current evidence points to an aberrant signal being conveyed. Importantly, although we have implied unidirectionality in this model, there are likely bidirectional processes (such as those posited by predictive coding frameworks) that are as yet unknown and need to be explored in further studies.

On the basis of the evidence presented above—specifically, the importance of social context, involvement of insula and motor cortex, lack of differential involvement of primary auditory cortex and high incidence of behavioural mimicry in misophonia—we outline a simplified neural model for a social cognitive perspective of misophonia in figure 1. From this perspective, auditory stimuli are conveyed through lower auditory pathways to motor regions, as well as higher levels of the auditory system, such as superior temporal sulcus, transforming the signal into a motor representation. Indeed, increased connectivity is evident between the auditory system and motor regions in participants with misophonia [14]. Depending on the context of the auditory stimulus, this aberrant signal is then communicated to the insula, as evidenced by increased functional connectivity between motor regions and insula [14], causing increased activation in this region [28]. Relevant to this, neuroimaging data show that the anterior insula is engaged when countering a mirrored action, i.e. either deliberately or unintentionally performing an opposite imitative movement [57], suggestive of an error signal being conveyed to this region. The insula then in turn may communicate this signal to the amygdala, resulting in an emotional response, as well as areas involved in autonomic reactions, such as periaqueductal gray (a midbrain structure involved in autonomic function), causing changes in physiology (e.g. heart rate, galvanic skin response) as previously observed [28,58]. This neurobiology of misophonia involving a perception—action network highlights a potential explanation for the high incidence of mimicry, which may involve either unconscious or deliberate imitation, in an attempt to provide relief to the person experiencing misophonia and thus inhibiting a hyperactive insula.

A potential therapeutic implication of a model incorporating the social cognitive aspects of misophonia would be to consider whether the underlying neurobiological or physiological responses to trigger sounds are changed by an altered context of the stimulus, in a similar manner to the premise of Samermit *et al*. [17]. That is, if a person with misophonia experiences a trigger sound re-framed in a different context, does this diminish the severity of their response? In the context of a social cognitive model of misophonia, one may assume that training participants to re-frame the context of the stimulus could provide relief and act as a treatment strategy. Relevant to this, in a study of non-misophonia participants, galvanic skin responses—an indicator of stress—caused by ‘nails screeching’ on a blackboard were diminished and auditory stimuli were rated less unpleasant if participants were under the cued impression that the stimulus was from a piece of contemporary music [15]. Additionally, any therapies based around a model that places a greater emphasis on the context of the stimulus, rather than the stimulus *per se*, would likely be generalizable to the visual analogue of misophonia, i.e. misokinesia, of which to-date—to our knowledge—only two dedicated studies exist [59,60]. Indeed, preliminary evidence suggesting some efficacy from cognitive behavioural therapy for misophonia is based on the premise that context is a critical component of the aversive response [61–67].

A number of untested hypotheses are proposed under the current model. The precise neural mechanisms of action perception in general are still under considerable debate, although the role of mirror neurons underlying imitation—defined as neurons that fire both when observing and executing an action—is supported even by the most prominent critics of the functional importance of mirror neurons [45,68]. Further exploration of the role of the perception–action system in mimicry in people with and without misophonia will help to inform the underlying neural basis of mimicry, both as it relates to this condition and also in a broader social cognitive framework. Furthermore, it is likely that refinements of this model—or indeed any other models—will need to attempt to account for heterogeneity of trigger types in the condition. As is evident with other conditions, such as tinnitus, a homogeneous approach that ignores the non-uniformity of a particular condition can often result in overlooking certain treatment strategies for particular subtypes, owing to these same strategies not working for other subtypes of the particular condition [29,69]. With such a strategy, treatments can then be developed to attempt to alleviate misophonia on an individualized basis and hopefully minimize variation in treatment response.

Consideration should also be given to whether misophonia reflects more generalized hypersensitivity to stimuli in other sensory domains, at least in a subset of sufferers. This aspect has been suggested as a potential possibility by others previously [70,71] and there are validated scales available to examine sensitivity across multiple sensory domains [72]. It is worth noting that this does not negate the idea of misophonia being viewed within a social cognitive context. Rather, if indeed misophonia reflects heightened sensitivity in other sensory domains, it would seem plausible that those with a more generalized sensitivity may show a greater disposition for mimicry, for example, and may mimic not necessarily to provide relief but rather because they are predisposed to do so on an involuntary basis, owing to a heightened social awareness. In support of this idea, in our most recent large online study [41], we did find that while many mimicked on a voluntary basis to provide relief, some others with misophonia mimicked in an automatic, involuntary fashion.

There are some evident limitations to the model at present and further research is needed to determine the precise roles of hyperactivated brain regions in misophonia, such as motor cortex and insula. One approach would be to determine specificity of activation in motor cortex—that is, if the current model is correct, given the presence of the motor homunculus [73,74], one would assume that someone with a dominant hand trigger would show greater activation in hand motor cortex, while someone with a dominant orofacial trigger would show greater activity in the corresponding orofacial motor cortex. As mentioned earlier, the precise role of the anterior insula in misophonia should also be elucidated in future studies, which could include determining whether there are differences in the activation of this region in misophonia beyond those driven by trigger sounds (for example, differences in interoceptive awareness). Additionally, there are some circumstances in which people with misophonia are triggered that do not appear to be within the context of social cognition, such as based on a ticking clock or animal sounds [22]. At present, we can only speculate how these may fit within our model outlined here, but it is possible that the specific social context in which someone has previously heard these sounds may have resulted in associations between those sounds and the social context, creating an association that results in the sound becoming a triggering stimulus. This idea would likely involve neural mechanisms related to associative memory, as it is well known that strong associations between seemingly unrelated events can create lasting memories and links between these events (for a review, see [75]).

There are further limitations with existing neuroimaging studies that need to be addressed to determine how well this model is supported by evidence. For example, the existing studies do not account for other comorbid factors, such as hyperacusis or anxiety. Thus, it is unclear to what extent the imaging findings may be mediated by some of these extraneous factors. This also has implications for treatment strategies. Based on another auditory pathology (tinnitus), comorbidities appear to affect responses to treatment (e.g. [76]). Therefore, future studies should account for these comorbidities as best as possible, to fully understand the nature of misophonia and its interactions with other conditions.

## Data Availability

This article has no additional data.
